# Mutation screening of *SPTLC1* and* SPTLC2* in amyotrophic lateral sclerosis

**DOI:** 10.1186/s40246-023-00479-3

**Published:** 2023-03-25

**Authors:** Chunyu Li, Yanbing Hou, Qianqian Wei, Junyu Lin, Zheng Jiang, Qirui Jiang, Tianmi Yang, Yi Xiao, Jingxuan Huang, Yangfan Cheng, Ruwei Ou, Kuncheng Liu, Xueping Chen, Wei Song, Bi Zhao, Ying Wu, Bei Cao, Yongping Chen, Huifang Shang

**Affiliations:** Department of Neurology, Laboratory of Neurodegenerative Disorders, National Clinical Research Center for Geriatrics, West China Hospital, Sichuan University, No.37, Guoxue Lane, Chengdu, 610041 Sichuan China

**Keywords:** Amyotrophic lateral sclerosis, Rare variant, *SPTLC1*, *SPTLC2*

## Abstract

**Background:**

Recently, several rare variants of *SPTLC1* were identified as disease cause for juvenile amyotrophic lateral sclerosis (ALS) by disrupting the normal homeostatic regulation of serine palmitoyltransferase (SPT). However, further exploration of the rare variants in large cohorts was still necessary. Meanwhile, *SPTLC2* plays a similar role as *SPTLC1* in the SPT function.

**Methods:**

To explore the genetic role of *SPTLC1* and *SPTLC2* in ALS, we analyzed the rare protein-coding variants in 2011 patients with ALS and 3298 controls from the Chinese population with whole exome sequencing. Fisher’s exact test was performed between each variant and disease risk, while at gene level over-representation of rare variants in patients was examined with optimized sequence kernel association test (SKAT-O).

**Results:**

Totally 33 rare variants with minor allele frequency < 0.01 were identified, including 17 in *SPTLC1* and 16 in *SPTLC2*. One adult-onset patient carried the variant p.E406K (*SPTLC1*) which was reported in previous study. Additionally, three adult-onset patients carried variants in the same amino acids as the variants identified in previous studies (p.Y509C, p.S331T, and p.R239Q in *SPTLC1*). At gene level, rare variants of *SPTLC1* and *STPLC2* were not enriched in patients*.*

**Conclusion:**

These results broadened the variant spectrum of *SPTLC1* and *SPTLC2* in ALS, and paved the way for future research. Further replication was still needed to explore the genetic role of *SPTLC1* in ALS.

**Supplementary Information:**

The online version contains supplementary material available at 10.1186/s40246-023-00479-3.

## Introduction

Amyotrophic lateral sclerosis (ALS) is a severe motor neuron disease characterized by the degeneration of upper and lower motor neurons [1]. Evidence from clinical and basic research has suggested multiple causes of ALS, including the essential role of genetic components. Since the first ALS causative gene *SOD1* was identified in 1993, mutations in more than 50 genes have been identified as potential cause for ALS [2]. However, the majority of patients still have no identifiable genetic causes, indicating more risk genes are to be explored.

Recently, several rare variants (p.A20S, p.Y23F, p.L39del, p.S331Y, and p.F40_S41del) of gene serine palmitoyltransferase long chain base subunit 1 (*SPTLC1*) were identified as the disease cause for juvenile ALS [3, 4]. *SPTLC1* encodes a subunit of serine palmitoyltransferase (SPT), and has been implicated in another neurological disorder hereditary sensory and autonomic neuropathy type 1 (HSAN1). Most of the variants of *SPTLC1* for ALS were in exon 2, while the variants for HSAN1 were more divergently distributed [4]. However, the findings have not been validated in other cohorts, especially in the population from different ethnic backgrounds. In addition, *SPTLC2* was also a major genetic cause of HSAN1, and played a similar role as *SPTLC1* in the SPT function [5].

In this context, we aimed to explore the rare protein-coding variants of *SPTLC1* and *SPTLC2* in a large Chinese ALS cohort. We identified one patient who carried the variant p.E406K reported in previous study, and three rare variants in the same amino acids as the variants reported in previous studies (p.Y509C, p.S331T, p.R239Q in *SPTLC1*).

## Materials and methods

### Participants

Totally 2011 ALS patients of Chinese ancestry were recruited from the Department of Neurology of West China Hospital of Sichuan University (Additional file 1: Table S1). Two specialized neurologists diagnosed the patients according to the El Escorial criteria. All the patients have signed informed consent. The control group of the Taiwanese Schizophrenia Trio Collection was used as controls (*n* = 3298) [6], who were unaffected parents of probands diagnosed with schizophrenia.

### Whole exome sequencing

For the patients, genomic DNA was extracted from peripheral blood mononuclear cells using standard phenol–chloroform procedures [7], and the NanoDrop instrument was used for purity and quantitation evaluation. Whole exome sequencing was conducted routinely on the Illumina NovaSeq 6000 system following the manufacturer’s instructions. Reads were mapped to the reference genome (UCSC hg19) by the Burrows-Wheeler Aligner (BWA) software to get the original mapping result in BAM format, and GATK software was utilized to get the mutation file in vcf format with common pipelines. For controls from the Taiwanese Schizophrenia Trio Collection, DNA collected from blood was sequenced on Illumina HiSeq sequencers for whole exome [6].

### Variant analysis

The rare variants which met the following criteria were analyzed: (1) minor allele frequency (MAF) was lower than 0.01; (2) variants were annotated as missense, splice donor, splice acceptor, start-lost, stop-gained, stop-loss or frameshift substitution by ANNOVAR; (3) the variant was either heterozygote or homozygote. Allelic association analysis was performed with standard Fisher’s exact test using default parameters. Bonferroni-corrected *P* value below 0.05 was considered as significant. The summary data of the East Asian population from gnomAD v2.1.1 were used as population controls.

### Gene-based burden analysis

Gene-based rare variant burden analysis was conducted to evaluate the aggregate association of rare variants with ALS using the optimized sequence kernel association test (SKAT-O). Sex and the first three principal components derived from population structure using GCTA v1.93.1 were adjusted. We categorized variants into rare (MAF < 0.01) and ultra-rare variants (MAF < 0.001). For each category, we tested the association for all rare variants and rare damaging variants, which were predicted as damaging or pathogenic by at least five out of ten *in-silico* prediction tools, including SIFT, Polyphen2 HDIV, Polyphen2 HVAR, LRT, Mutation Taster, Mutation Assessor, FATHMM, MetaSVM, MetaLR, CADD (Additional file 1: Table S2).

## Results

We analyzed the rare variants of *SPTLC1* and *SPTLC2* in 2011 patients with ALS of Chinese ancestry. The average age at onset (SD) was 54.32 (11.76) with a sex ratio of 1.45 (male/female: 1190/821). A total of 33 rare variants (MAF < 0.01) were identified, including 17 in *SPTLC1* and 16 in *SPTLC2*. These variants comprised 30 missense, 1 stop-gain and 2 frameshift substitution variants, among which 24 were predicted as damaging (Table 1, Additional file 1: Table S2). Thirty of these variants were ultra-rare (MAF < 0.001), and 25 were absent in controls (Table 1). One patient carried the same variant p.E406K as reported in another ALS patient of Italian ancestry from previous study [3]. This patient developed bulbar dysfunction as the initial symptoms at forties. Additionally, three adult-onset patients carried rare variants in the same amino acids as the variants reported previously, namely p.Y509C, p.S331T, and p.R239Q in *SPTLC1*, corresponding to p.Y509X, p.S331Y, and p.R239W [3] (Table 2). All the three variants were predicted as damaging by at least five prediction tools. Furthermore, two novel rare variants p.Q229R (*SPTLC1*) and p.G435V (*SPTLC2*) were detected in two patients with juvenile ALS. One patient carried variant p.T409M (*SPTLC2*), which was suggested to cause HSAN1 [8]. This variant was predicted as damaging by eight prediction tools and has a GERP score of 5.65, suggesting high evolutionary conservation. Clinically, this patient developed ALS at the fifties, presenting with weakness in the proximal upper limb. He also had some autonomic dysfunction, like hyperhidrosis, frequent urination, and heat intolerance. No variant was associated with the risk of ALS by Fisher’s exact test (Table 1), and rare variants at gene level were not enriched in the patients (Additional file 1: Table S3).

**Table 1:** Rare variants of *SPTLC1* and *SPTLC2* identified in patients with amyotrophic lateral sclerosis

Genomic position	Rsid	Annotation	hgvs_c	hgvs_p	gnomAD as control (*N* = 9977)			Normal control (*N* = 3298)			Dam
					Control MAF	*P*	OR (95% CI)	Control MAF	*P*	OR (95% CI)	
9:94794611	rs1288792833	Missense	c.A1526G	p.Y509C	0 (0/12136)	0.249	Inf (0.08-Inf)	0 (0/6596)	0.379	Inf (0.04-Inf)	
9:94794623	rs189582528	Missense	c.A1514C	p.K505T	7.70E-5 (1/12980)	0.417	3.23 (0.04–252.87)	0 (0/6596)	0.379	Inf (0.04-Inf)	
9:94794633	n.a	Missense	c.A1504G	p.I502V	0 (0/19954)	0.168	Inf (0.13-Inf)	0 (0/6596)	0.379	Inf (0.04-Inf)	
9:94800568	rs750753414	Missense	c.G1216A	p.E406K	1.09E-4 (2/18394)	0.447	2.29 (0.04–43.94)	1.52E-4 (1/6596)	1.000	1.64 (0.02–128.64)	D
9:94800570	rs567541925	Missense	c.G1214A	p.R405H	0 (0/19952)	0.168	Inf (0.13-Inf)	0 (0/6596)	0.379	Inf (0.04-Inf)	D
9:94800579	rs576468489	Missense	c.C1205G	p.T402S	0 (0/19954)	0.168	Inf (0.13-Inf)	0 (0/6596)	0.379	Inf (0.04-Inf)	
9:94800586	n.a	Stopgain	c.G1198T	p.E400X	0 (0/19954)	0.168	Inf (0.13-Inf)	0 (0/6596)	0.379	Inf (0.04-Inf)	D
9:94809456	rs1587914537	Missense	c.C1079T	p.P360L	0 (0/19954)	0.168	Inf (0.13-Inf)	0 (0/6596)	0.379	Inf (0.04-Inf)	D
9:94809510	n.a	Frameshift substitution	c.1025delins TCGTTACC	p.P342Lfs*12	0 (0/19954)	0.168	Inf (0.13-Inf)	0 (0/6596)	0.379	Inf (0.04-Inf)	D
9:94809544	n.a	Missense	c.T991A	p.S331T	0 (0/19954)	0.168	Inf (0.13-Inf)	0 (0/6596)	0.379	Inf (0.04-Inf)	D
9:94817751	rs773121449	Missense	c.G716A	p.R239Q	0 (0/18384)	0.180	Inf (0.12-Inf)	0 (0/6596)	0.379	Inf (0.04-Inf)	D
9:94821465	rs1236408812	Missense	c.A686G	p.Q229R	0 (0/19954)	0.168	Inf (0.13-Inf)	0 (0/6596)	0.379	Inf (0.04-Inf)	D
9:94821469	rs1290542896	Missense	c.G682A	p.D228N	0 (0/19954)	0.168	Inf (0.13-Inf)	0 (0/6596)	0.379	Inf (0.04-Inf)	D
9:94821511	rs781435924	Missense	c.A640G	p.M214V	5.45E-5 (1/18360)	0.327	4.57 (0.06–357.35)	0 (0/6596)	0.379	Inf (0.04-Inf)	
9:94830356	rs45461899	Missense	c.G452A	p.R151H	2.46E-3 (49/19904)	0.476	0.71 (0.27–1.57)	4.09E-3 (27/6596)	0.050	0.42 (0.16–1.00)	D
9:94841727	rs181424155	Missense	c.C50T	p.P17L	6.41E-4 (1/1560)	1.000	0.78 (0.04–45.79)	0 (0/6596)	0.143	Inf (0.31-Inf)	
9:94843164	rs752029386	Missense	c.C342A	p.N114K	0 (0/18392)	0.032	Inf (0.86-Inf)	0 (0/6596)	0.143	Inf (0.31-Inf)	D
14:77978635	rs1484082947	Missense	c.G1681A	p.E561K	0 (0/19954)	0.168	Inf (0.13-Inf)	0 (0/6596)	0.379	Inf (0.04-Inf)	D
14:77978695	rs746495804	Missense	c.C1621T	p.R541C	0 (0/18392)	0.179	Inf (0.12-Inf)	0 (0/6596)	0.379	Inf (0.04-Inf)	D
14:77984475	n.a	Missense	c.G1475A	p.G492D	0 (0/19954)	0.168	Inf (0.13-Inf)	0 (0/6596)	0.379	Inf (0.04-Inf)	D
14:77987811	n.a	Missense	c.C1417G	p.L473V	0 (0/19954)	0.168	Inf (0.13-Inf)	0 (0/6596)	0.379	Inf (0.04-Inf)	D
14:77987924	rs879253951	Missense	c.G1304T	p.G435V	0 (0/19954)	0.168	Inf (0.13-Inf)	0 (0/6596)	0.379	Inf (0.04-Inf)	D
14:78018481	rs557959879	Missense	c.A1261G	p.T421A	5.44E-5 (1/18384)	0.327	4.57 (0.06–357.82)	1.52E-4 (1/6596)	1.000	1.64 (0.02–128.64)	D
14:78018516	rs368357970	Missense	c.C1226T	p.T409M	0 (0/19936)	0.168	Inf (0.13-Inf)	0 (0/6596)	0.379	Inf (0.04-Inf)	D
14:78023441	rs375502150	Missense	c.A899G	p.Q300R	0 (0/19954)	0.168	Inf (0.13-Inf)	0 (0/6596)	0.379	Inf (0.04-Inf)	D
14:78036791	n.a	Missense	c.C692G	p.A231G	0 (0/19954)	0.168	Inf (0.13-Inf)	0 (0/6596)	0.379	Inf (0.04-Inf)	D
14:78045345	rs749262868	Missense	c.G435T	p.R145S	8.15E-4 (15/18394)	0.753	0.61 (0.07–2.62)	1.67E-3 (11/6596)	0.150	0.30 (0.03–1.37)	
14:78045373	rs760762454	Missense	c.G407A	p.R136Q	0 (0/18392)	0.179	Inf (0.12-Inf)	0 (0/6596)	0.379	Inf (0.04-Inf)	D
14:78063530	n.a	Missense	c.A326G	p.K109R	0 (0/19954)	0.168	Inf (0.13-Inf)	0 (0/6596)	0.379	Inf (0.04-Inf)	
14:78063569	rs2079852183	Missense	c.T287C	p.I96T	0 (0/19954)	0.168	Inf (0.13-Inf)	0 (0/6596)	0.379	Inf (0.04-Inf)	D
14:78063573	n.a	Frameshift substitution	c.283delinsGA	p.R95Efs*3	0 (0/19954)	0.168	Inf (0.13-Inf)	0 (0/6596)	0.379	Inf (0.04-Inf)	D
14:78063717	n.a	Missense	c.C139T	p.H47Y	0 (0/19954)	0.168	Inf (0.13-Inf)	0 (0/6596)	0.379	Inf (0.04-Inf)	D
14:78082850	rs1017782894	Missense	c.G73C	p.V25L	1.92E-3 (23/11960)	0.271	0.52 (0.13–1.51)	7.58E-4 (5/6596)	0.738	1.31 (0.26–6.10)	

*MAF* Minor allele frequency; *n.a.* Not available; Variants with MAF < 0.01 were considered rare; *P* and OR values were obtained using Fisher’s exact test implemented in R 3.6.2 with default parameters; RefSeq accession numbers: *SPTLC1*, NM_001281303; *SPTLC2*, NM_004863. Genomic position was based on GRCh37. Dam denotes whether the variant is protein-truncating variant or missense variant which is predicted as damaging by at least five out of ten prediction tools, with D indicating that the variant is damaging

**Table 2:** Clinical features of patients with specific variants in *SPTLC1* and *SPTLC2*

Clinical feature	Patient 1	Patient 2	Patient 3	Patient 4	Patient 5	Patient 6
Gene change	p.S331T	p.R239Q	p.E406K	p.Q229R	p.T409M	p.G435V
Sex	Male	Female	Female	Male	Male	Female
Fasciculation	Yes	No	No	Yes	Yes	No
Spasticit	No	No	No	No	No	No
Drug exposure	No	No	No	No	No	No
Smoking	Now	No	No	No	Now	No
Drinking	Used to drink	No	No	Used to drink	No	No
Sensory	Normal	Normal	Normal	Normal	Normal	Normal
Toxin	Pesticide	No	No	No	Pesticide	No
Onset site	Proximal lower limb	Distal upper limb	Bulbar	Proximal upper limb	Proximal upper limb	Proximal lower limb
Onset symptom	Fatigue	Fatigue	Dysarthria	Fatigue	Fatigue	Fatigue
Tongue myoclonus	No	Yes	Yes	Yes	Yes	No
Tongue atrophy	No	Yes	Yes	Yes	Yes	No
Choking	No	Yes	Yes	No	No	No
Dysphagia	No	Yes	Yes	No	No	No
Dysarthria	No	Yes	Yes	No	No	No
Pharyngeal reflex	Hyporeflexia	Hyperreflexia	Disappear	Hyporeflexia	Disappear	Normal

## Discussion

In the current study, we explored the rare variants of *SPTLC1* and *SPTLC2* in a large Chinese ALS cohort. We identified the variant p.E406K (*SPTLC1*) reported in previous study, and three variants in the same amino acids as the variants reported in previous studies (p.Y509C, p.S331T, and p.R239Q). These finding broadened the current variant spectrum of *SPTLC1* and *SPTLC2* in ALS, and provided a foundation for further research.

*SPTLC1* and *SPTLC2* encode subunits 1 and 2 of SPT, an enzyme that catalyzes the biosynthesis of serine and palmitoyl CoA. Upregulation of SPT was suggested to play a role in apoptosis, suggesting its potential role in neurodegeneration. Variants in *SPTLC1* and *SPTLC2* were originally reported in patients with HSAN1, a rare peripheral neuropathy typically characterized by a slow and progressive sensory loss and the formation of perforating ulcers at the feet and hands. The variants could induce a permanent shift in the substrate preference from L-serine to L-alanine, which resulted in the pathological formation of atypical and neurotoxic 1-deoxy-sphingolipids [8]. Recently, several variants in *SPTLC1* were identified as the disease cause for juvenile ALS. Compared with the variants for HSAN1, the variants for ALS disrupt the normal homeostatic regulation of SPT by ORMDL proteins, resulting in unregulated SPT activity and elevated levels of canonical SPT products [4]. Considering that alterations in SPT activity have been linked to neurodegeneration, and the close relation between *SPTLC1* and *SPTLC2*, we also analyzed the rare protein-coding variants of *SPTLC2*.

In the current cohort, two patients with juvenile ALS carried variants p.Q229R (*SPTLC1*) and p.G435V (*SPTLC2*), both of which were absent in controls. WES suggested the two patients did not carry pathogenic variants in known ALS-related genes (https://alsod.ac.uk/). The patient with p.Q229R developed ALS at the age of 25, presenting with weakness in the proximal upper limb. He had tongue fasiculation, tongue atrophy and hyporeflexia. The variant p.G435V in *SPTLC2* was suggested to cause HSAN1 [9]. This variant could lead to reduced effector-cytokine production and reduction of T cell proliferation, which was associated with a significant increase in apoptosis [9].

One patient carried the same variant p.E406K (*SPTLC1*) as reported in previous study [3]. This variant is rare in both the European and Chinese populations (MAF < 0.0001) based on data from gnomAD. Detection of the rare variant in patients from different ancestries suggested its potential role in ALS. However, considering the limited sample size, further replication was still warranted. Meanwhile, this variant was reported as potential cause for HSAN1 in ClinVar, though the clinical significance of this variant was still uncertain. Meanwhile, one ALS patient in our dataset carried p.T409M (*SPTLC2*), which was suggested to cause HSAN1 as well [8]. This variant was absent in controls, and predicted as damaging by eight prediction tools. Similarly, the rare variant p.S331Y (*SPTLC1*) was detected in both ALS and HSAN1 patients, suggesting potential overlapping pathogenesis between the two disorders. Incomplete penetrance or other modifier genes might contribute to the heterogeneous phenotypes for the same variant. Further exploration of the functional effect of these variants might provide additional insight.

In addition, three variants (p.Y509C, p.S331T, p.R239Q) in the same amino acids as previous variants were identified. Among the three variants, p.S331T and p.R239Q were absent in controls, while p.Y509C was ultra-rare (MAF < 0.001). All the three variants were predicted as damaging by at least 5 prediction tools, and had high GERP scores (5.25, 4.52 and 5.3). All the three patients were with adult-onset ALS, suggesting potential association between specific variants of *SPTLC1* and adult-onset ALS, which needs further exploration.

Though several rare variants of *SPCLC1* and *SPTLC2* were detected in patients with ALS, whether these variants were disease cause of these patients was unknown. Meanwhile, we did not identify an association between the rare variants and disease risk at both variant and gene levels, disapproving the genetic role of these two genes in ALS to some extent. However, the results should be interpreted with caution since the sample size was relatively small, and an association is hard to detect for rare variants in case–control designed studies, especially in the context of etiological heterogeneity and incomplete penetrance. Additionally, the rare variants of *SPTLC1* were detected in juvenile ALS in the original study, while in the current cohort most of the patients were adult-onset. Therefore, further replication in juvenile ALS with larger sample size is still necessary.

In conclusion, we systematically analyzed the rare variants of *SPLTC1* and *SPTLC2* in ALS with association analyses at variant and gene levels. Our work broadened the current variant spectrum of *SPTLC1* and *SPTLC2* in ALS, and provided a foundation for future research on these two genes in ALS.

## Supplementary Information

**Additional file 1: Table S1.** Demographic data for the enrolled cohorts. **Table S2.** In-silico pathogenicity predictions for rare variants in *SPTLC1* and *SPTLC2*. **Table S3.** Enrichment analysis of rare variants in *SPTLC1* and *SPTLC2* in amyotrophic lateral sclerosis.

## Data Availability

The genotype and phenotype data of the Taiwanese Schizophrenia Trio Collection were applied from dbGap (accession number phs001196.v1.p1). The dataset supporting the conclusions of this article is included within the article and its additional file.
