# Supplementary material for: Mutation screening of SPTLC1 and SPTLC2 in amyotrophic lateral sclerosis
Source: Hum Genomics. 2023 Mar 25;17:28. doi: 10.1186/s40246-023-00479-3 (PMC10040122; doi:10.1186/s40246-023-00479-3)
Supplement: Supplementary file 1 — Additional file 1: Table S1. Demographic data for the enrolled cohorts. Table S2. In-silico pathogenicity predictions for rare variants in SPTLC1 and SPTLC2. Table S3. Enrichment analysis of rare variants in SPTLC1 and SPTLC2 in amyotrophic lateral sclerosis. [file 40246_2023_479_MOESM1_ESM.docx]

**Table S1. Demographic data for the enrolled cohorts.**

| **Group** | **total No.** | **Age at onset (SD)** | **Male/Female ratio** |
| --- | --- | --- | --- |
| ALS cohort | 2011 | 54.32 (11.76) | 1.45 (1190/821) |
| control cohort | 3298 | Not applicable | 1.04 (1681/1617) |

No., the number of individuals; ALS, amyotrophic lateral sclerosis.

**Table S2. In-silico pathogenicity predictions for rare variants in *SPTLC1* and *SPTLC2*.**

| **hgvs_c** | **hgvs_p** | **GERP++** | **Variant-effect predictions software (scores)** | | | | | | | | | |
| --- | --- | --- | --- | --- | --- | --- | --- | --- | --- | --- | --- | --- |
|  |  |  | **SIFT** | **Polyphen2 HDIV** | **Polyphen2 HVAR** | **LRT** | **Mutation Taster** | **Mutation Assessor** | **FATHMM** | **MetaSVM** | **MetaLR** | **CADD** |
|  |  |  |  |  |  |  |  |  |  |  |  |  |
| c.A1526G | p.Y509C | n.a. | n.a. | n.a. | n.a. | n.a. | n.a. | n.a. | n.a. | n.a. | n.a. | n.a. |
|  |  |  | n.a. | n.a. | n.a. | n.a. | n.a. | n.a. | n.a. | n.a. | n.a. | n.a. |
| c.A1514C | p.K505T | n.a. | n.a. | n.a. | n.a. | n.a. | n.a. | n.a. | n.a. | n.a. | n.a. | n.a. |
|  |  |  | n.a. | n.a. | n.a. | n.a. | n.a. | n.a. | n.a. | n.a. | n.a. | n.a. |
| c.A1504G | p.I502V | n.a. | n.a. | n.a. | n.a. | n.a. | n.a. | n.a. | n.a. | n.a. | n.a. | n.a. |
|  |  |  | n.a. | n.a. | n.a. | n.a. | n.a. | n.a. | n.a. | n.a. | n.a. | n.a. |
| c.G1216A | p.E406K | 5.3 | 0.232 | 0.139 | 0.044 | 0 | 1 | 1.065 | -3.6 | 0.201 | 0.706 | 23.8 |
|  |  |  | T | B | B | D | D | L | D | D | D | D |
| c.G1214A | p.R405H | 4.38 | 0.561 | 1 | 0.964 | 0 | 1 | 1.245 | -3.61 | 0.509 | 0.825 | 27.2 |
|  |  |  | T | D | D | D | D | L | D | D | D | D |
| c.C1205G | p.T402S | 5.3 | 0.605 | 0.002 | 0.01 | 0 | 0.973 | -0.175 | -2.61 | -0.513 | 0.38 | 14.92 |
|  |  |  | T | B | B | D | D | N | D | T | T | D |
| c.G1198T | p.E400X | 4.33 | n.a. | n.a. | n.a. | 0.156 | 1 | n.a. | n.a. | n.a. | n.a. | 39 |
|  |  |  | n.a. | n.a. | n.a. | N | A | n.a. | n.a. | n.a. | n.a. | D |
| c.C1079T | p.P360L | 5.35 | 0.106 | 0.18 | 0.168 | 0 | 1 | 1.155 | -2.67 | 0.132 | 0.648 | 27.6 |
|  |  |  | T | B | B | D | D | L | D | D | D | D |
| c.1025delins  TCGTTACC | p.P342L  fs*12 | n.a. | n.a. | n.a. | n.a. | n.a. | n.a. | n.a. | n.a. | n.a. | n.a. | n.a. |
|  |  |  | n.a. | n.a. | n.a. | n.a. | n.a. | n.a. | n.a. | n.a. | n.a. | n.a. |
| c.T991A | p.S331T | 5.25 | 0.041 | 0.218 | 0.259 | 0 | 1 | 1.57 | -2.62 | 0.335 | 0.686 | 24.8 |
|  |  |  | D | B | B | D | D | L | D | D | D | D |
| c.G716A | p.R239Q | 4.52 | 0.003 | 1 | 0.977 | 0 | 1 | 3.395 | -3.63 | 1.086 | 0.93 | 35 |
|  |  |  | D | D | D | D | D | M | D | D | D | D |
| c.A686G | p.Q229R | 5.09 | 0.386 | 0.003 | 0.01 | 0 | 1 | 0.14 | -3.54 | -0.003 | 0.624 | 14.57 |
|  |  |  | T | B | B | D | D | N | D | T | D | D |
| c.G682A | p.D228N | 5.09 | 0.038 | 0.289 | 0.262 | 0 | 1 | 2.49 | -2.58 | 0.404 | 0.724 | 33 |
|  |  |  | D | B | B | D | D | M | D | D | D | D |
| c.A640G | p.M214V | 0.162 | 0.562 | 0.001 | 0.02 | 0 | 1 | 1.125 | -2.64 | -0.511 | 0.511 | 6.92 |
|  |  |  | T | B | B | N | D | L | D | T | D | T |
| c.G452A | p.R151H | 4.14 | 0.03 | 0.028 | 0.104 | 0 | 1 | 3.295 | -2.76 | 0.552 | 0.777 | 25.8 |
|  |  |  | D | B | B | D | D | M | D | D | D | D |
| c.C50T | p.P17L | n.a. | n.a. | n.a. | n.a. | n.a. | n.a. | n.a. | n.a. | n.a. | n.a. | n.a. |
|  |  |  | n.a. | n.a. | n.a. | n.a. | n.a. | n.a. | n.a. | n.a. | n.a. | n.a. |
| c.C342A | p.N114K | 1.16 | 0.143 | 0.473 | 0.213 | 0 | 0.999 | 1.71 | -3.64 | 0.146 | 0.715 | 17.77 |
|  |  |  | T | P | B | D | D | L | D | D | D | D |
| c.G1681A | p.E561K | 5.56 | 0.027 | 0.956 | 0.899 | 0 | 1 | 0 | -3.68 | 0.751 | 0.788 | 26.5 |
|  |  |  | D | P | P | D | D | N | D | D | D | D |
| c.C1621T | p.R541C | 5.56 | 0 | 1 | 0.959 | 0 | 1 | 0 | -3.73 | 0.936 | 0.831 | 26.6 |
|  |  |  | D | D | D | D | D | N | D | D | D | D |
| c.G1475A | p.G492D | 5.59 | 0.002 | 0.999 | 0.984 | 0 | 1 | 3.185 | -2.98 | 0.997 | 0.903 | 31 |
|  |  |  | D | D | D | D | D | M | D | D | D | D |
| c.C1417G | p.L473V | 3.69 | 0.19 | 0.345 | 0.31 | 0 | 1 | 0.34 | -3.74 | 0.075 | 0.692 | 23 |
|  |  |  | T | B | B | N | D | N | D | D | D | D |
| c.G1304T | p.G435V | 5.73 | 0 | 1 | 0.997 | 0 | 1 | 3.655 | -2.46 | 0.986 | 0.887 | 27.9 |
|  |  |  | D | D | D | D | D | H | D | D | D | D |
| c.A1261G | p.T421A | 5.64 | 0.219 | 0.02 | 0.02 | 0 | 1 | 0.26 | -3.14 | -0.143 | 0.56 | 17.46 |
|  |  |  | T | B | B | D | D | N | D | T | D | D |
| c.C1226T | p.T409M | 5.65 | 0.115 | 0.89 | 0.418 | 0 | 1 | 3.54 | -2.79 | 0.827 | 0.818 | 24 |
|  |  |  | T | P | B | D | D | H | D | D | D | D |
| c.A899G | p.Q300R | 5.05 | 0.021 | 0.932 | 0.708 | 0 | 1 | 2.095 | -2.78 | 0.663 | 0.765 | 26.4 |
|  |  |  | D | P | P | D | D | M | D | D | D | D |
| c.C692G | p.A231G | 5.49 | 0.36 | 0.277 | 0.392 | 0 | 1 | 2.665 | -2.74 | -0.011 | 0.509 | 24.9 |
|  |  |  | T | B | B | D | D | M | D | T | D | D |
| c.G435T | p.R145S | -1.07 | 0.321 | 0.004 | 0.017 | 0.002 | 0.985 | 0.805 | -3.5 | -0.415 | 0.599 | 14.98 |
|  |  |  | T | B | B | N | N | L | D | T | D | D |
| c.G407A | p.R136Q | 5.65 | 0.051 | 0.761 | 0.148 | 0 | 1 | 2.255 | -0.48 | -0.293 | 0.323 | 25.1 |
|  |  |  | T | P | B | D | D | M | T | T | T | D |
| c.A326G | p.K109R | 4.88 | 0.113 | 0.054 | 0.043 | 0 | 1 | 1.935 | -0.46 | -0.707 | 0.241 | 17.48 |
|  |  |  | T | B | B | D | D | M | T | T | T | D |
| c.T287C | p.I96T | 4.88 | 0.19 | 0.913 | 0.638 | 0 | 1 | 1.34 | -0.57 | -0.437 | 0.332 | 24.1 |
|  |  |  | T | P | P | D | D | L | T | T | T | D |
| c.283  delinsGA | p.R95Efs*3 | n.a. | n.a. | n.a. | n.a. | n.a. | n.a. | n.a. | n.a. | n.a. | n.a. | n.a. |
|  |  |  | n.a. | n.a. | n.a. | n.a. | n.a. | n.a. | n.a. | n.a. | n.a. | n.a. |
| c.C139T | p.H47Y | 4.88 | 0.219 | 0.641 | 0.084 | 0 | 0.987 | 1.5 | -3.6 | 0.442 | 0.7 | 18 |
|  |  |  | T | P | B | D | D | L | D | D | D | D |
| c.G73C | p.V25L | 2.11 | 1 | 0 | 0 | 0.069 | 0.992 | -0.695 | -3.51 | -0.535 | 0.451 | 11.14 |
|  |  |  | T | B | B | N | N | N | D | T | T | D |

T, tolerated; D, damaging or disease causing; P, probably pathogenic; N, neutral; M, medium; L, low; B, benign; n.a., not available. GERP, genomic evolutionary rate profiling; SIFT, sorting intolerant from tolerant; PolyPhen2 HDIV, polymorphism phenotyping version 2 human diversity; PolyPhen2 HVAR, polymorphism phenotyping version 2 human variation; LRT, likelihood ratio test; FATHMM, functional analysis through hidden Markov models; SVM, support vector machine; LR, logistic regression; CADD, combined annotation dependent depletion. Pathogenicity prediction was obtained using ANNOVAR.

**Table S3. Enrichment analysis of rare variants in *SPTLC1* and *SPTLC2* in amyotrophic lateral sclerosis.**

| **variant group** | **Gene** | **Rare variant** | | | **Ultra-rare variant** | | |
| --- | --- | --- | --- | --- | --- | --- | --- |
|  |  | **Case** | **Control** | **P** | **Case** | **Control** | **P** |
| all variants | *SPTLC1* | 18 | 59 | 0.69 | 18 | 17 | 0.69 |
|  | *SPTLC2* | 21 | 34 | 0.09 | 19 | 11 | 0.27 |
| damaging variants | *SPTLC1* | 11 | 40 | 0.60 | 11 | 13 | 0.60 |
|  | *SPTLC2* | 14 | 16 | 0.16 | 14 | 9 | 0.17 |

Rare variant denotes variant with minor allele frequency < 0.01; Ultra-rare variant denotes variant with minor allele frequency < 0.001. P values were calculated using SKAT-O (optimized sequence kernel association test). Damaging variants denotes rare variants which were predicted as damaging or pathogenic by at least five out of ten in-silico prediction tools. Case and Control denotes the number of alleles detected in patients and controls.
